# Correction to ‘Constructing A Core Competency Evaluation Index System for Incontinence Specialist Nurses: A Delphi Study’

**DOI:** 10.1002/nop2.71052

**Published:** 2026-11-11

Tian, X. M. , Y. Z. Chen , S. M. Xie , H. M. Shao , Y. Y. Li , and J. Liu . 2026. “Constructing a Core Competency Evaluation Index System for Incontinence Specialist Nurses: A Delphi Study.” Nursing Open 13, no. 6: e70617. 10.1002/nop2.70617.PMC1324056342201911

The article type has been changed from ‘Discursive Paper’ to ‘Empirical Research Quantitative’.

We apologize for this error.

